# Oliceridine effectively attenuates fentanyl-induced cough during general anesthesia induction

**DOI:** 10.3389/fmed.2026.1786137

**Published:** 2026-05-14

**Authors:** Ru-Yi Xing, Wen-Yi Gong, Chen-Guang Li, Ilyar Mamtili, Shuang-Xiong Zhu, Wen-Jun Zhou, Bing-Xing Li, Jie Cao, Xiao-Fang Yue, Kun Fan

**Affiliations:** 1 Department of Anesthesiology, Shanghai Sixth People's Hospital Affiliated to Shanghai Jiao Tong University School of Medicine, Shanghai, China; 2 Department of Anesthesiology, Shanghai Xuhui Central Hospital, Shanghai, China; 3 Department of Anesthesiology, The First People's Hospital of Tianshui, Tianshui, Gansu, China; 4 Department of Cardiovascular Surgery, The First People's Hospital of Tianshui, Tianshui, Gansu, China; 5 Department of Anesthesiology, Yan’an Hospital Affiliated to Kunming Medical University, Kunming, Yunnan, China; 6 Department of Neurology, Shanghai Sixth People's Hospital Affiliated to Shanghai Jiao Tong University School of Medicine, Shanghai, China

**Keywords:** cough, fentanyl, general anesthesia, induction, oliceridine

## Abstract

**Objective:**

Fentanyl-induced cough (FIC) is a common adverse event during anesthesia induction with a high incidence and may result in serious clinical complications. Although our clinical observations suggest that oliceridine attenuates FIC, the available evidence remains limited. This study was designed to assess the prophylactic efficacy of oliceridine against FIC and to characterize its peri-induction safety profile.

**Methods:**

A total of 168 adult surgical patients with American Society of Anesthesiologists physical status I–III scheduled for general anesthesia were randomized to receive either oliceridine 2 mg (OF group) or normal saline (SF group) prior to fentanyl administration. The primary outcome was the incidence of cough within 1 min following fentanyl injection. Secondary outcomes included cough severity, vital sign changes, and the incidence of adverse events.

**Results:**

No patients in the OF group experienced FIC, compared to 58.33% in the SF group (*p* < 0.001). Cough severity in the SF group was classified as mild (17.86%), moderate (20.24%), and severe (20.24%). Vital signs did not show statistically significant changes from before to after oliceridine injection within each group, and no significant between-group differences were identified. The incidence of adverse events was low in both groups, with no significant between-group differences.

**Conclusion:**

Pretreatment with 2 mg oliceridine effectively reduces the incidence of FIC without increasing significant additional risks, providing a rationale and safe approach for anesthetic induction.

**Clinical trial registration:**

https://www.chictr.org.cn/showproj.html?proj=272947, identifier ChiCTR2500105221.

## 1 Introduction

Fentanyl is a classic potent opioid, first synthesized in 1960, widely utilized for clinical anesthesia and perioperative analgesia (1). However, reflex cough is a common adverse event associated with intravenous fentanyl administration. Reported incidence rates of fentanyl-induced cough (FIC) vary significantly, ranging from 21.8 to 75%, depending on dosage and administration techniques (2–9). Cough during induction may elevate systemic blood pressure, intraocular pressure, intracranial pressure, and intra-abdominal pressure to varying degrees and may cause airway obstruction (10). In some cases, this cough can be spasmodic, explosive (11), and potentially life-threatening (12), significantly compromising patient safety during induction.

Various pharmacological and non-pharmacological interventions have been proposed to manage opioid-induced cough (OIC); however, their effectiveness and safety remain controversial (10). Therefore, there is an urgent clinical need for novel, more effective, and safer strategies for preventing FIC.

Oliceridine, approved by the U.S. Food and Drug Administration (FDA) in 2020, is a novel intravenous analgesic that acts as a G protein-biased agonist at the *μ*-opioid receptor with approximately five-fold the analgesic potency of morphine, primarily indicated for managing acute pain in adults (13). Our clinical observations suggest that oliceridine effectively inhibits FIC, though evidence remains sparse. Thus, we designed this prospective, randomized, double-blind, placebo-controlled clinical study to evaluate the efficacy of oliceridine in preventing FIC during general anesthesia induction.

## 2 Methods

### 2.1 Study design and ethics

This prospective, single-center, randomized, double-blind, placebo-controlled trial adhered to the ethical standards outlined in the Declaration of Helsinki. Approval was granted by the Ethics Committee of the First People’s Hospital of Tianshui (Approval No. 2025-08; Date: May 8, 2025), and the trial was registered in the Chinese Clinical Trial Registry (Registration No.: ChiCTR2500105221). This study was conducted and reported in accordance with the Consolidated Standards of Reporting Trials (CONSORT) guidelines. All participants or their legal representatives provided written informed consent prior to inclusion.

### 2.2 Enrollment

Initial patient enrollment was performed by distributing brochures in the surgical wards of the First People’s Hospital of Tianshui. Inclusion criteria were patients classified as American Society of Anesthesiologists (ASA) physical status I–III, aged 18–70 years, scheduled for general anesthesia. Exclusion criteria included allergies, sinus bradycardia, severe neurological, respiratory or cardiovascular diseases, anesthetic drug dependence, recent opioid use, hepatic or renal dysfunction, pregnancy, delivery, lactation, and patients with upper respiratory tract infections within 2 weeks prior to surgery that might lead to spontaneous coughing.

### 2.3 Randomization and blinding

Patients were randomized equally into Oliceridine-Fentanyl (OF) or Saline-Fentanyl (SF) groups using computer-generated random sequences.^1^ Allocation concealment was ensured by using sealed opaque sequentially numbered envelopes, managed by the research coordinator. On the day of surgery, envelopes were handed to a pharmacist who prepared the study medications without further involvement. The OF group received oliceridine 2 mg diluted with normal saline to a total volume of 5 mL, whereas the SF group received 5 mL normal saline. The investigational drugs were transferred into indistinguishable syringes and labeled sequentially as “Syringe 1” and “Syringe 2.” Once preparation was completed, the syringes were passed to the designated investigator for administration. The allocation envelopes were then resealed and returned to the research coordinator. All trial medications were kept at ambient temperature (20–22 °C) and administered within 10 min of preparation. Blinding was maintained for patients, research coordinators, administering investigators, and data analysts throughout the entire trial.

### 2.4 Anesthesia and research procedure

Patients were cannulated with a 22G intravenous (IV) catheter on the dorsum of the right forearm and continuously infused with a balanced crystalloid solution. Upon entry into the operating room, continuous monitoring of heart rate, noninvasive arterial pressure, and oxygen saturation was established and sustained until completion of the surgical procedure.

Patients received intravenous injections of either oliceridine 2 mg or an equivalent volume of 0.9% normal saline, administered over 2 s. A standardized flush with 2 mL of normal saline over 2 s was injected immediately after drug injection to ensure complete drug delivery and avoid residual medication in the tubing. During injection, the intravenous carrier fluid was temporarily stopped via the three-way stopcock. Subsequently, fentanyl at 4 μg/kg was administered intravenously over 2 s. Fentanyl was prepared at a concentration of 50 μg/mL, with clearly labeled syringes indicating drug name and concentration. The injection speed was standardized by having all injections administered by the same anesthesiologist, and the duration of drug administration was strictly controlled using a stopwatch. A blinded, rigorously trained research coordinator evaluated and recorded the incidence and severity of reflex cough within 1 min following fentanyl administration. No other drugs were administered before the observed medications. Following completion of the observation period, or in cases where oxygen saturation fell below 90%, patients received intravenous propofol (1–1.5 mg/kg) and rocuronium (0.6 mg/kg), and positive-pressure ventilation was initiated. General anesthesia was maintained at the anesthesiologist’s discretion.

### 2.5 Outcome assessment

All study outcomes were evaluated and recorded by the research coordinator. The primary outcome was the incidence of cough following fentanyl administration. Fentanyl-induced cough was defined as an audible cough accompanied by expiratory effort occurring after fentanyl administration, without obvious inspiratory prolongation or stridor. Secondary outcomes included: (1) the number of patients experiencing different severities of cough, classified as mild (1–2 coughs), moderate (3–5 coughs), or severe (>5 coughs) (14); (2) systolic blood pressure (SBP), diastolic blood pressure (DBP), heart rate (HR), and peripheral oxygen saturation (SpO_2_) measured before administration of oliceridine or normal saline (T0) and 2 min after fentanyl injection (T1); and (3) other adverse effects regarding oliceridine and fentanyl, including chest wall rigidity, oxygen desaturation (SpO_2_ < 90%), respiratory apnea (defined as cessation of respiratory movement for more than 15 s), nausea and vomiting.

### 2.6 Sample size calculation

Sample size estimation was conducted using PASS 11 software (NCSS, LLC, Kaysville, Utah, USA), based on the primary endpoint—the incidence of cough following fentanyl administration. According to previous data (10), the baseline incidence of FIC was approximately 49.7%. Assuming that pretreatment with oliceridine would result in a 50% reduction in this incidence, a minimum of 76 patients per group was required to achieve 90% statistical power at a two-sided significance level (*α* = 0.05). To account for an anticipated 10% dropout rate, 84 patients were ultimately enrolled in each group.

### 2.7 Statistical analysis

Statistical analyses were conducted using IBM SPSS Statistics version 22 (IBM Corp., Armonk, NY, USA). Normality of continuous variables was evaluated using the Kolmogorov–Smirnov test, and homogeneity of variance was assessed via Levene’s test. Data with normal distribution and equal variance were expressed as mean ± standard deviation (SD) and compared using the independent samples *t*-test for intergroup comparisons and paired *t*-tests for within-group comparisons. Non-normally distributed variables were expressed as median [interquartile range] and analyzed using the Mann–Whitney *U* tests and Wilcoxon signed-rank test, respectively. Categorical variables were reported as counts (percentages) and analyzed using Pearson’s *χ*^2^ test or Fisher’s exact test, as appropriate. Effect sizes were reported as absolute risk reduction (ARR) and number needed to treat (NNT), with corresponding 95% confidence interval (CI). Relative risks (RR) were additionally calculated using the Haldane–Anscombe correction for zero-cell counts. Between-arm comparisons of ordinal categorical variables were not estimable due to zero-cell counts in the treatment group; therefore, only descriptive distributions were presented for the control group. A two-sided *p*-value of less than 0.05 was considered statistically significant.

## 3 Results

**Figure 1:**
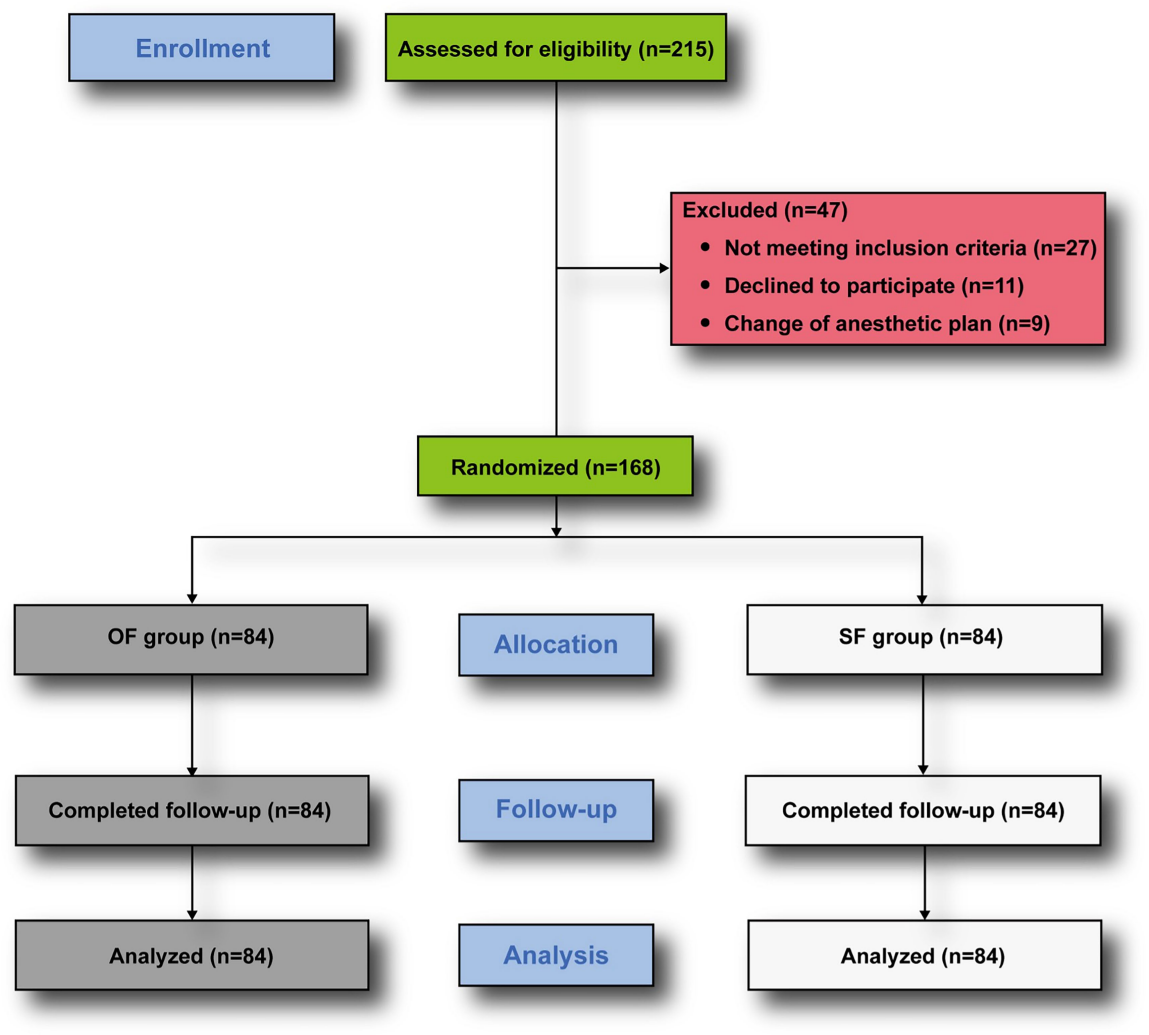
Consolidated standards of reporting trials flow diagram.

A total of 215 patients initially met the preliminary eligibility criteria, of which 168 patients met all inclusion and exclusion criteria and were randomly allocated evenly into the SF and OF groups. Forty-seven patients were excluded due to not meeting inclusion criteria, refusal to participate, or changes in anesthetic method. All randomized patients completed the study and were included in the final analysis; no patients withdrew from the study (Figure 1). No statistically significant differences were found in demographic characteristics or the amount of fentanyl administered during induction between the two groups (Table 1).

**Table 1:** Demographic characteristics and total dose of fentanyl.

Variable	OF group (*n* = 84)	SF group (*n* = 84)	*p*-value
Age (years)	49.14 ± 13.79	51.58 ± 11.88	0.221^a^
Gender, male/female	45/39	37/47	0.217^b^
Weight (kg)	62.74 ± 10.11	63.86 ± 10.99	0.493^a^
Height (cm)	164.26 ± 7.94	162.68 ± 8.13	0.203^a^
ASA class (1/2/3)	18/41/25	16/43/25	0.921^b^
Total dose of fentanyl (μg)	250.95 ± 40.44	255.43 ± 43.95	0.493^a^

Data were presented as mean ± SD, or number of patients. ^a^independent samples *t*-test; ^b^Pearson’s *χ*^2^ test; ASA, American Society of Anesthesiologists physical status; OF group, Oliceridine group; SF group, Saline group.

No patients in the OF group experienced FIC, whereas the incidence in the SF group was 58.33%, with mild, moderate, and severe cough occurring in 17.86, 20.24, and 20.24% of patients, respectively. The incidence of cough was significantly lower in the OF group compared with the SF group (0% vs. 58.33%, *p* < 0.001), corresponding to an absolute risk reduction (ARR) of 0.58 (95% CI: 0.46–0.68), a number needed to treat (NNT) of 2 (95% CI: 2–3), and a corrected relative risk (RR) of 0.01 (95% CI: 0.00–0.16) (Table 2).

**Table 2:** Incidence and severity of fentanyl-induced cough and adverse events after treatment in both groups.

Variable	OF group (*n* = 84)	SF group (*n* = 84)	Estimate (95% CI)	*p*-value
Incidence of cough, *n* (%)	0 (0.00)	49 (58.33)	ARR: 0.58 (0.46–0.68); NNT: 2 (2–3); RR: 0.01 (0.00–0.16)^*^	<0.001^a^
Severity of cough, *n* (%)			NA	NA
Mild	0 (0.00)	15 (17.86)		
Moderate	0 (0.00)	17 (20.24)		
Severe	0 (0.00)	17 (20.24)		
Adverse events, *n* (%)				
Chest wall rigidity	0 (0.00)	1 (1.19)	NA	1.000^a^
Hypoxemia	0 (0.00)	0 (0.00)	NA	NA
Nausea and vomiting	1 (1.19)	0 (0.00)	NA	1.000^a^

Data were presented as number (%). ARR, absolute risk reduction; NNT, number needed to treat; RR: relative risk; CI, confidence interval; NA, not applicable (non-estimable comparisons); OF group, Oliceridine group; SF group, Saline group; ^a^Fisher’s exact test; ^*^Haldane–Anscombe correction for zero-cell counts.

There were no significant differences between the OF and SF groups regarding SBP, DBP, HR, and SpO_2_ at either T0 or T1 (Table 3). Additionally, there were no significant changes in SBP, DBP, HR, and SpO_2_ before and after oliceridine injection in either group. In the SF group, one patient developed chest wall rigidity, and the symptoms resolved after administration of a muscle relaxant. In the OF group, one patient experienced nausea without vomiting, which required no intervention. The between-group differences in the incidence of chest wall rigidity and nausea and vomiting were not statistically significant. No hypoxemia was observed in either group (Table 2).

**Table 3:** Vital sign changes in both groups.

Variable	OF group (*n* = 84)	SF group (*n* = 84)	*p*-value
SBP (mmHg)			
T0	115.79 ± 16.34	111.96 ± 12.61	0.091^a^
T1	111.21 ± 14.02	110.63 ± 15.29	0.800^a^
*p*-value	0.071^c^	0.529^c^	
DBP (mmHg)			
T0	72.27 ± 8.64	73.37 ± 8.97	0.418^a^
T1	74.61 ± 14.00	74.95 ± 11.15	0.860^a^
*p*-value	0.161^c^	0.331^c^	
HR (beats/min)			
T0	81.80 ± 10.21	82.02 ± 8.93	0.897^a^
T1	79.17 ± 9.83	80.71 ± 12.61	0.376^a^
*p*-value	0.096^c^	0.389^c^	
SPO_2_ (%)			
T0	98 [97, 99]	98 [97, 99]	0.923^b^
T1	99 [97, 99]	99 [98, 100]	0.295^b^
*p*-value	0.144^d^	0.880^d^	

Data were presented as mean ± SD, or median [IQR]. SBP, systolic blood pressure; DBP, diastolic blood pressure; HR, heart rate; SpO_2_, peripheral oxygen saturation; T0, immediately prior to administration of oliceridine or normal saline; T1, 2 min after fentanyl injection; OF group, Oliceridine group; SF group, Saline group; IQR, interquartile range; ^a^independent samples *t*-tests; ^b^Mann–Whitney *U* tests; ^c^paired *t*-tests; ^d^Wilcoxon signed-rank tests.

## 4 Discussion

This study identified a FIC incidence of 58%, aligning with previously reported data. Notably, pretreatment with oliceridine was associated with a marked reduction in FIC, with an ARR of 0.58 (95% CI: 0.45–0.68) and a NNT of 2 (95% CI: 2–3), indicating a clinically meaningful effect, without an apparent increase in adverse events. Although the corrected RR was 0.01 (95% CI: 0.00–0.16), also suggesting a substantial reduction in the incidence of FIC; however, the estimate may be unstable due to the presence of zero events. To our knowledge, this finding has rarely been reported in the publications.

Cough frequently occurs following fentanyl administration during general anesthesia induction. The incidence of FIC is influenced by various factors, including drug dosage and concentration, administration sequence, injection speed and site, individual physical condition, age, gender, weight, medical history, smoking history, and genetic factors (15). The exact pathophysiological mechanism underlying FIC remains incompletely understood, and several hypotheses have been proposed. Fentanyl injection may disrupt the sympathetic-vagal balance, increasing vagal tone and thus triggering cough (16). Additionally, fentanyl activates pulmonary vascular vagal C-fiber receptors, mediating pulmonary chemical reflexes and causing bronchoconstriction, thereby inducing cough (17). Furthermore, fentanyl may decrease chest wall compliance, leading to sudden vocal cord adduction or supraglottic airway obstruction, resulting in cough (18). Histamine and neuropeptide release mediated by presynaptic *μ*-opioid receptors following fentanyl injection may also induce cough (12).

The precise mechanisms by which oliceridine pretreatment prevents fentanyl-induced cough cannot be determined from the present data and remain speculative. Several hypothetical mechanisms can be proposed based on the pharmacological properties of oliceridine. First, as a G protein-biased μ-opioid receptor agonist (19), oliceridine may preferentially bind to μ-opioid receptors, thereby blocking fentanyl’s access to these receptors and preventing cough induction. Second, oliceridine may deplete neurotransmitters within pulmonary vagal C-fibers, attenuating the afferent signaling responsible for triggering cough. Third, the concurrent administration of oliceridine and fentanyl may produce synergistic effects via dual μ-receptor activation, stabilizing autonomic tone by modulating both sympathetic and parasympathetic activity. Lastly, oliceridine may enhance centrally mediated muscle relaxation induced by fentanyl, reduce vocal cord tension, and preserve respiratory muscle tone, collectively decreasing airway hyperresponsiveness and further suppressing the cough reflex. These mechanisms are not directly supported by the present data and should be considered as theoretical considerations and directions for future research.

A variety of pharmacologic agents—including butorphanol, lidocaine, ketamine, dexmedetomidine, low-dose fentanyl, propofol, dezocine, dexamethasone, and magnesium sulfate—have been evaluated for the prevention of OIC (20–22). However, most of these interventions fail to fully suppress the cough reflex and are associated with undesirable side effects (21, 23–25). Moreover, previous literature consistently indicates that the majority of premedication strategies are insufficient in completely eliminating opioid-induced cough during the induction phase (20–22). These limitations have limited their clinical utility and raised concerns regarding safety during anesthetic induction. In contrast, the present study demonstrates that oliceridine pretreatment nearly eliminated FIC without clinically significant side effects, highlighting its promise as a safer and more effective prophylactic strategy in clinical anesthesia, particularly for patients with elevated intracranial pressure, increased intraocular pressure, bronchial asthma, or those requiring awake intubation.

This study has several limitations. First, the optimal dose of oliceridine for the prevention of FIC was not investigated. Second, the limited sample size may have precluded the detection of rare adverse events. Third, the study did not include a direct comparison with other antitussive interventions, thereby limiting conclusions regarding the most effective preventive strategy. Fourth, the use of a rapid, high-dose fentanyl bolus (4 μg/kg) in this study may have increased the baseline incidence of cough in the control group, thereby amplifying the between-group difference. This finding may be applicable only under the specific study conditions. Fifth, patients at high risk of cough—such as those with airway disease, elevated intracranial pressure, increased intraocular pressure, or severe cardiopulmonary disease—were not included in this study, which limits the generalizability of the findings. Future studies should address these limitations to more comprehensively evaluate the efficacy and safety profile of oliceridine in mitigating FIC.

## 5 Conclusion

In conclusion, pretreatment with 2 mg oliceridine significantly reduces the incidence of FIC without notable adverse effects, providing a rationale and safe approach for anesthetic induction. Oliceridine holds potential clinical value in preventing FIC.

## Data Availability

The raw data supporting the conclusions of this article will be made available by the authors, without undue reservation.

## Footnotes

1
http://www.random.org
